# Feasibility of Patient- and Caregiver-Reported Outcome Measures in Incurable Cancer: Implications for Early Palliative Care Communication

**DOI:** 10.3390/jcm15218449

**Published:** 2026-10-30

**Authors:** Hsien Seow, Daryl Bainbridge, Rosalyn A. Juergens, Oren Levine, Courtney Coschi, Sarah Mah, Karen Zhang, Zhimeng Jia, Jeffrey Myers, Anish K. Arora, Nadia Incardona, Leah Steinberg

**Affiliations:** 1 Department of Oncology, McMaster University, Hamilton, ON L8S 4L8, Canada; 2 Temmy Latner Centre for Palliative Care, Sinai Health Systems, Toronto, ON M5T 3L9, Canada; 3 Department of Family and Community Medicine, University of Toronto, Toronto, ON M5S 1A1, Canada; 4 Department of Health Sciences, Wilfrid Laurier University, Waterloo, ON N2L 3C5, Canada

**Keywords:** palliative care, communication, caregivers, neoplasms, patient-reported outcome measures

## Abstract

**Background:** Advances in cancer treatment mean many individuals with incurable cancer live for extended periods, creating opportunities for early integration of palliative care. High-quality communication about illness trajectory, patient values, and goals is a core mechanism through which early palliative care improves patient and family outcomes. Yet patient- and caregiver-reported outcomes reflecting these domains are rarely measured early in the illness trajectory. To address this gap, we tested the feasibility of collecting validated patient- (and caregiver-) reported outcome measures (PROMs) capturing domains central to serious illness communication (SIC) and more broadly, an early palliative approach to care. **Methods:** In this cross-sectional study at a regional cancer centre, patients with incurable cancer and their caregivers completed an online survey within three days of a routine oncology visit. PROMs included Feeling Heard and Understood, Herth Hope Index, Illness Understanding, Hospital Anxiety and Depression Scale, and the Preparedness for Caregiving Scale (caregivers only). Feasibility metrics included recruitment, completion rates, data completeness, and perceived importance of PROMs. **Results:** Seventy-two patients and 65 caregivers completed the survey (83% and 95% of those approached). All prespecified feasibility metrics were met, with minimal missing data and high willingness to repeat surveys (>93%). Most respondents (>79%) rated each PROM as important. PROM scores were a secondary descriptive outcome, with hope and feeling heard and understood rated relatively high. For illness understanding, 17% of patients and 9% of caregivers believed the cancer would be cured completely. Caregiver emotional burden was substantial: 49% reported elevated anxiety compared with 31% of patients. **Conclusions:** Collecting PROMs reflecting communication quality, illness understanding, hope, anxiety and depression, and caregiving preparedness early in the incurable cancer trajectory appeared feasible within the conditions of this study and suggests a practical way to evaluate outcomes central to SIC, which is a key part of an early palliative approach to care.

## 1. Introduction

Advances in treatment for metastatic and incurable cancers have extended survival, sometimes by several years, resulting in prolonged periods during which patients and families require support in understanding the illness, navigating uncertainty, and preparing for future decisions [1,2]. Early palliative care aims to meet these needs by providing timely, person-centred communication, emotional support, pain and symptom management, and guidance across the illness trajectory [3]. Decades of research demonstrate that palliative care improves patient-reported symptoms, satisfaction, and care experiences [4,5,6,7,8]. In particular, evidence shows that early integration of palliative care, often within weeks of diagnosis, further enhances patient-reported outcomes [9,10,11,12,13].

Despite this evidence, palliative care is frequently introduced late, with many patients receiving services only in the final weeks of life [14,15]. An early palliative approach to care has been conceptualized as encompassing communication regarding illness and prognosis, symptom assessment and management, coping support, and regular follow-up; considered “early” when initiated prior to the end-of-life period, which is often defined as the last six months before death [16,17,18]. High-quality communication is central to initiating an early palliative approach, yet patients with serious illness often report worsening communication experiences and greater uncertainty as their condition progresses [19]. As the main care providers for patients with advanced cancer, oncologists are well positioned to introduce palliative approaches earlier, but discomfort and hesitation in initiating difficult conversations often limit timely integration [20,21]. Serious illness communication (SIC) provides a structured approach to discussing prognosis, values, goals, and preferences, and is a key mechanism through which an early palliative approach achieves its effects.

Although numerous SIC and palliative care communication training programs exist, most evaluations rely on clinician-reported outcomes such as satisfaction or perceived competence [22]. Few studies assess real-world patient- or caregiver-reported outcomes, and a recent systematic review found that fewer than one-third of communication training trials included patient-reported outcome measures (PROMs), with no consistent outcome set identified [22,23,24]. This gap limits the ability to determine whether palliative care and communication training meaningfully improves patient and caregiver experiences, especially early in an incurable cancer trajectory.

PROMs offer a way to capture outcomes central to SIC competencies, including illness understanding, hope, emotional distress, feeling heard and understood, and caregiver preparedness [25,26,27]. PROMs are often considered the gold standard for evaluating healthcare interventions [25,28]. However, collecting PROMs among seriously ill patients presents methodological challenges, including concerns about acceptability due to illness burden, potential for high missing data, and uncertainty about the value of questionnaires [17,28,29,30,31]. Therefore, establishing feasible and acceptable approaches to collecting PROMs early in the incurable cancer trajectory is essential.

The objective of this study was to test the feasibility of collecting patient- and caregiver-reported outcome measures among individuals with advanced incurable cancer following routine oncology visits. These PROMs reflect domains central to SIC and are also relevant to a palliative approach to care and routine oncology care. The primary aim was to determine whether PROMs could be feasibly and acceptably collected in this population. A secondary aim was to describe the PROM scores related to communication quality, illness understanding, hope, emotional distress, and caregiving preparedness early in the incurable cancer trajectory.

## 2. Materials and Methods

### 2.1. Study Design

This study was a cross-sectional study of patients with incurable cancer and their caregivers.

### 2.2. Population and Eligibility Criteria

Participants were recruited from oncology clinics at a large regional cancer centre in Ontario, Canada. Participating clinicians included four oncology providers (medical and radiation oncologists) who practised within breast, lung, gastro-intestinal, and gynecologic oncology subspecialties. We set a pragmatic sample size of a minimum of 50 patients based on the oncologists’ estimated caseloads over a month and an expectation that 50% of patients approached for the study would decline.

Patients were eligible if they had an incurable cancer diagnosis and were not expected to die within the next six months, had been informed of their prognosis, and had attended at least one prior visit with the participating (the patient’s main) oncologist since being informed of this prognosis. The only additional eligibility criterion was the ability to complete the survey in English. All consecutive eligible patients were approached to minimize selection bias. Family caregivers accompanying eligible patients were also invited to participate independently using a caregiver-specific survey. Caregivers not present at the appointment were not eligible to participate as we could not verify this role.

### 2.3. Patient Recruitment

We used a consecutive sampling method. Within a three-week window, oncologists determined incurability, self-screened patients scheduled for a clinic visit in advance, flagged those eligible for the study, and approached all eligible patients during their visit to invite them to complete the survey. The oncologists maintained a screening log of all patients they screened, the outcome of the approach, and whether the patient attended the visit with a caregiver. The log was used to ensure that patients were not approached twice and was collected at recruitment completion by the analyst (DB) to provide data on study accrual. The analyst communicated with the oncologists on an ongoing basis (weekly minimum) as a study reminder and to obtain recruitment updates.

Patients and the accompanying caregivers (if applicable) were provided a study information sheet and a postcard containing links to the patient- and caregiver-specific versions of the online survey. A paper version of the survey and stamped return envelope were available upon request. The survey was anonymous to reduce social desirability bias in responses, and patient and caregiver participants each received a $50 (CAD) electronic gift card upon completion. A $50 incentive was deemed appropriate to encourage study participation (and completion of open-text items) in consideration of the physical and mental burden those living with advanced cancer may face. Data collection occurred between September 2025 and March 2026.

### 2.4. Data Collection

The oncologists tracked the number of patients and caregivers approached. Postcards were uniquely numbered to confirm the number of patients approached and the number who completed the survey. Participants were asked to complete the survey within three days of the clinic visit. Surveys were hosted on McMaster University’s LimeSurvey platform (version 6, ON Canada) and required approximately 20 min to complete. Upon survey completion, participants entered the unique postcard identifier and their contact information on a separate form to enable gift card distribution, while preserving anonymity.

### 2.5. Outcome Measures

The primary outcome was feasibility, assessed using recruitment rates, survey completion, and completeness of data collection. These metrics are summarised descriptively and were consistent with criteria previously used in a pilot trial of a SIC intervention [32]. We deemed a threshold of 60% of patients approached who agree to consider participating and two-thirds of those who complete a survey as reasonable given web-based surveys for patients average a response rate of around 50% [33] and many journals deem 60% as a minimum threshold for survey response rates [34,35]. For this population, given their illness trajectory and the sensitivity of illness discussions, we estimated that about half of participants who completed a survey would be willing to complete it again a month later and that only about a third of patients would be comfortable having a conversation with their oncologist recorded. This last metric was exploratory to assess the potential of collecting objective data from the clinical encounter that could be used to corroborate PROMs.

Secondary outcomes included a set of patient- (and caregiver-) reported outcome measures (PROMs). The selection of PROMs was guided by Street et al.’s communication outcomes framework [36] and a recent environmental scan of patient- and caregiver-reported measures relevant to SIC [37]. A panel of 16 health care providers and/or researchers with expertise in SIC derived a shortlist of relevant, commonly used PROMs identified in the environmental scan. The panel met over time to review potential PROMS and reduce the selection of measures to a minimum dataset through discussion and consensus. PROMS were selected considering clinical relevance, response burden, construct validity, and coverage of the communication outcomes framework [36]. The PROMs, described in more detail below, were assessed from the respondent’s perspective, either as the patient or caregiver.

Illness Understanding: Seven items adapted from the Prognosis and Treatment Perceptions Questionnaire (PTPQ), which commonly consists of 13 questions [38]. We omitted items from the PTPQ that assess satisfaction with information, impacts of information provided, and the patient’s medical status. Rather, we focused this measure on the patient’s/caregiver’s illness understanding and perceived extent to which the patient’s preferences for information had been respected by their care team, concepts relevant to those with a recent prognosis of incurable cancer. Two items from the PTPQ, respectively assessing the respondent’s goal for treatment and what they believe this goal is of the oncologist, were modified to be more specific as to the likely outcome of the treatment and care plan, to reduce the number of response options, and to also elicit perceptions of the family’s understanding. The latter was intended to explore serious illness understanding concordance between the patient, the family, and the doctor, which is predicated on shared illness and prognostic awareness [39].

Feeling Heard and Understood Scale (FHU): This validated four-item measure assesses patient and caregiver perceptions of communication quality, including feeling heard and understood, therapeutic alliance, trust, and whole-person care [40,41]. Items are scored from 0 (“Not at all true”) to 4 (“Completely true”).

Herth Hope Index: The validated 12-item Herth Hope Index assesses perceptions of hope across domains such as outlook, inner strength, and sense of direction [42,43,44,45]. Items are scored from 1 to 4, with higher scores indicating greater hope.

Hospital Anxiety and Depression Scale (HADS): The HADS is a validated 14-item instrument measuring symptoms of anxiety and depression, scored on a 0–3 scale and categorized using standard cut-offs for the sum of all items (summary score: Normal = 0–7, Borderline = 8–10, or Abnormal = 11–21) [46,47].

Preparedness for Caregiving Scale (Caregivers only): Caregivers completed this validated 8-item measure assessing preparedness across practical and emotional caregiving domains [48,49,50,51]. Items are scored from 0 (“Not at all prepared”) to 4 (“Very well prepared”).

Two open-text items were included: (1) Indicate anything specific you would like to be better prepared for (Caregivers only) and (2) Comment about your experiences completing this survey.

### 2.6. Data Analysis

Quantitative data were analyzed descriptively using means, standard deviations, and frequencies to summarize feasibility metrics, participant characteristics, and PROM scores. PROM scores were calculated according to instrument guidelines: the total summary score for the HADS, overall score mean for the FHU, and both overall mean and summary score for the Herth Hope Index. No interpolation was performed for missing data. Quantitative analyses were conducted using IBM SPSS Statistics (Version 31).

A qualitative descriptive approach was used to conduct a structured, inductive thematic analysis on responses to open-text questions [52]. This analysis was conducted manually using Microsoft Excel (version 365). The analyst (DB), an experienced qualitative researcher, developed a systematic coding and thematic framework through the process of reading and re-reading the responses, generating initial codes, searching for initial themes among the codes, reviewing and refining themes by revisiting the data, and then defining and naming prominent themes. Final themes were reviewed and discussed among members of the research team until consensus was reached.

## 3. Results

**Figure 1:**
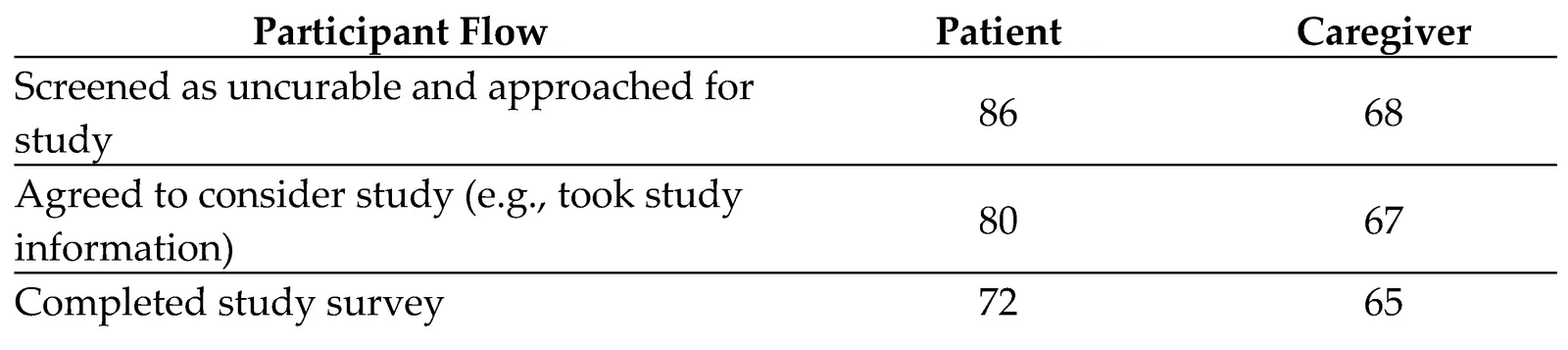
Participant flow diagram.

A total of 86 patients were approached, of whom 72 patients and 65 caregivers completed the survey (83% and 95% completion among those approached). Nearly all (99%) participants completed the survey online. Figure 1 presents the participant flow diagram. Average completion time was 13 min for patients and 15 min for caregivers. The sample was predominantly older adults (26% of patients younger than 60 years), with most patients living with a spouse or caregiver. Table 1 presents the patient and caregiver respondent characteristics. Eighty percent of caregivers and 87% of patients reported that they were born in this country (i.e., Canada). Over six different disease sites were represented. Lung cancer was the most common diagnosis (44%), followed by breast cancer (14%). Most patients (60%) had been diagnosed 1–5 years prior. Two-thirds of patients reported themselves as being “normal with no limitations” or “not their normal self but able to be up and about with fairly normal activities.” Caregiver respondents were often the patient’s married or common-law spouse (73.8%), followed by parent/parent-in-law (10.8%), son/daughter (7.7%), brother/sister (3.1%), or other (4.3%).

### 3.1. Feasibility Metrics

All feasibility thresholds were met, based on the available recruitment and completion data, see Table 2. Recruitment was high (93%), missing data were minimal (<0.1%), and nearly all respondents (93%) indicated willingness to complete the survey again at multiple future time points. Two-thirds of patients and 72% of caregivers were willing to have their oncology conversation recorded.

**Figure 2:**
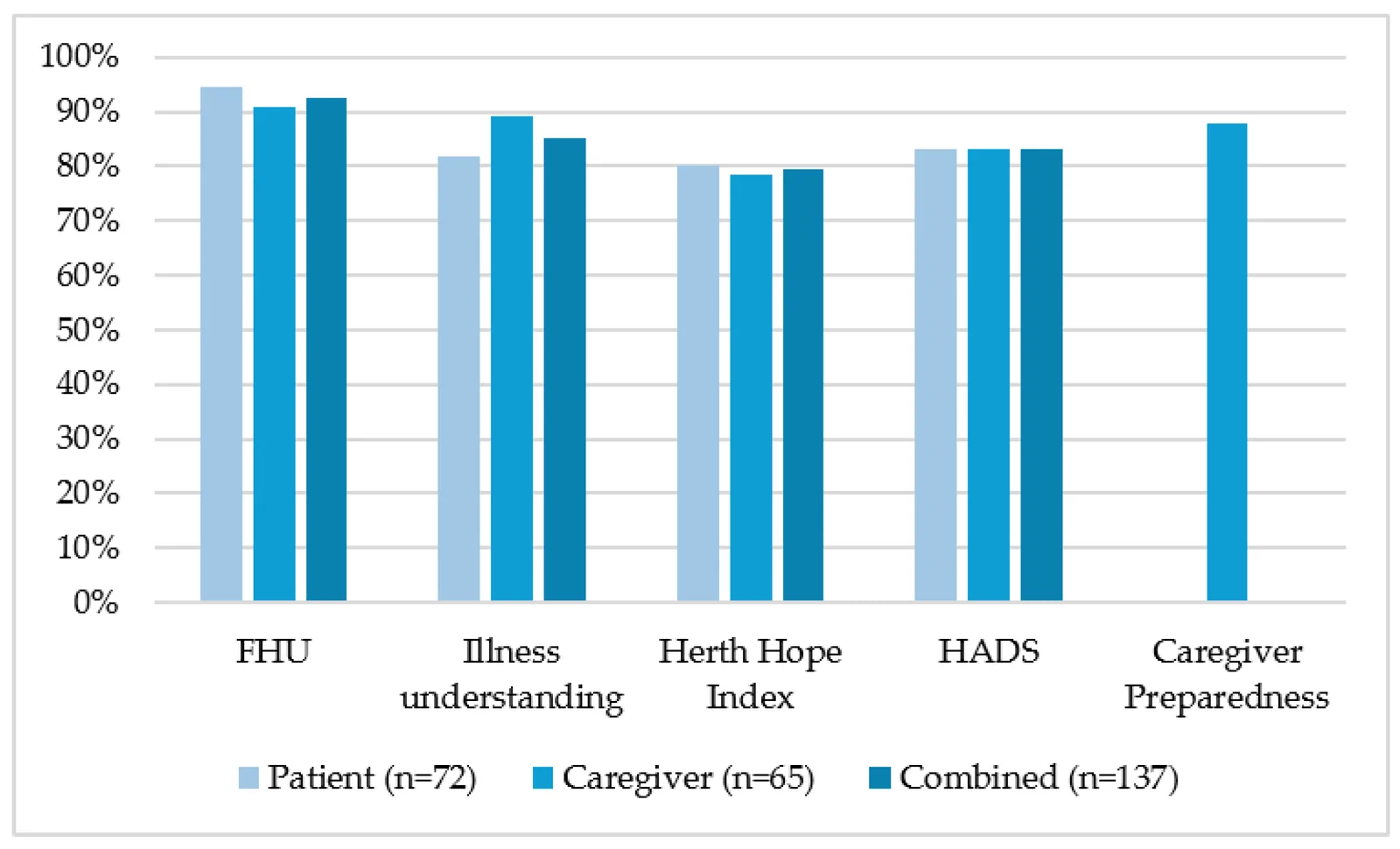
Perceived importance to respondent of each measure, rated as “important” or “very important”. FHU = Feeling Heard and Understood scale; HADS = Hospital Anxiety and Depression Scale.

Most respondents rated each PROM as “Important” or “Very Important”. Patient scores ranged from 82% for the Illness Understanding items to 94% for the Feeling Heard and Understood scale (FHU) (Figure 2). Caregivers tended to rate the Understanding items as more important (very important = 89%), and the Herth Hope Index as less important (very important = 79%), compared to patients. Twenty patients and about the same number of caregivers provided open-text comments about their experiences in completing the survey. Common themes included appreciating the opportunity to share their thoughts and feelings about the encounters with their oncologist and wishing that they could have done so earlier in the care journey.

### 3.2. Patient- and Caregiver-Reported Outcome Measures

The overall scores for each PROM are presented in Table 3. Feeling Heard and Understood scores were uniformly high for both patients and caregivers (mean 3.9/4.0) across all four FHU domains, indicating very high ratings of communication quality. Slight variation (7%) occurred between the FHU items in the proportion of respondents who answered “4—Completely true”.

High scores were also observed on the Herth Hope Index, with >90% of patients and caregivers agreeing or strongly agreeing with most items, including having a positive outlook toward life, short and/or long-range goals, a deep inner strength, and a sense of direction. About 22% of respondents felt alone and almost half (patients 52%, caregivers 48%) agreed that they were scared about the future. Both patients and caregivers had an overall mean score of 3.2/4.0 on the Herth Hope Index.

On the HADS, 31% of patients and 49% of caregivers had elevated anxiety (borderline or abnormal). Caregiver depression scores were also observed to be higher than patient scores (12% vs. 9% abnormal), suggesting greater caregiver emotional burden.

#### 3.2.1. Illness Understanding

Illness understanding responses, presented in Table 4, showed that nearly all participants felt their information preferences were respected by their oncologist (Item 1). Most patients (74%) and caregivers (85%) wanted to know as many details as possible relating to the cancer and its treatment (Item 3). Yet about 15% of patients and caregivers, respectively, wished they had more information about the prognosis (Item 2). While about half of patients (patients 49%, caregivers 37%) reported that they had discussed the prognosis either “often” or “very often” with the oncologist, about 12% of caregivers reported never discussing prognosis (Item 4). In terms of illness understanding, 17% of patients and 9% of caregivers believed that the patient’s cancer would likely be “cured completely” even though this was all incurable (Item 7). For patients and caregivers, there was slight discordance among those who believed that the cancer would be cured completely and whether they felt their family and doctors believed the same, with a few of these respondents tending to be more optimistic from their own perspective (Items 5–7).

#### 3.2.2. Caregiver Preparedness

On the Preparedness for Caregiving Scale, caregivers scored an overall mean of 2.6/4.0. The majority (52% to 75%) felt “Pretty well” or “Very well” prepared on most of the aspects mentioned in the scale (e.g., dealing with physical needs, setting up services, emergency needs, etc.), but less prepared to deal with the emotional aspects of this role, such as dealing with the stress of this role (35%) or keeping this activity pleasant (49%). On the single overall preparedness item, most caregivers were in the middle of the scale, 29.2% indicating that they are “Somewhat well prepared” and 53.8% reporting that they are “Pretty well prepared”.

Sixty percent (*n* = 38) of caregivers provided open-text comments specifying areas where they would like to be better prepared (mean response length 124 characters [20 to 454]). The central challenges in feeling prepared reported were summarized into five primary themes: Coping with the uncertainty of the illness; Preparing for the end stages; Impact on emotional and mental health; Facing anticipatory grief and loss; and Need for practical skills and medical knowledge. Caregivers identified uncertainty as a dominant concern, with the unpredictable nature of illness making it difficult to feel prepared or plan for the months to come.

“It is difficult to be prepared for the unpredictable. Even though we are aware there is no cure, we try to live in the present…” Caregiver 68 (spouse)

Caregivers expressed a strong need for clearer guidance around end-of-life care, particularly what to expect, how to manage transitions, and how to access hospice or palliative supports. The emotional burden of caregiving was prominent, with caregivers balancing their own mental health challenges while supporting the emotional needs of loved ones. Some caregivers reported experiencing grief in anticipation of the death of the patient, although these caregivers had accepted that nothing could be done about these feelings.

“Grief counseling would be of help. Even though the patient is living, there is still grief involved in watching and helping as best as able, knowing the outcome may not be a happy one.” Caregiver 58 (spouse)

In addition, caregivers reported needing more practical skills and medical knowledge, particularly for managing symptoms and responding to emergencies. Difficulties navigating healthcare and community services further contributed to stress, with many unsure how to access or coordinate supports. Overall, caregivers relayed the importance of proactive planning and receiving timely, structured information to better prepare for future changes.

## 4. Discussion

This study demonstrates that collecting patient- and caregiver-reported outcomes central to serious illness communication was feasible in the oncology practices we tested. All feasibility metrics were met, including recruitment, completion, data quality, and willingness to repeat surveys. Caregiver participation was particularly strong, addressing a persistent gap in SIC and palliative care research where caregiver perspectives are often underrepresented [53,54]. A small minority of patients reported spending “most of the day in bed” or “rarely out of bed”, underscoring that these PROMs can be collected earlier in a cancer journey. Ultimately, these findings support the feasibility of integrating PROMs early in the incurable cancer trajectory to capture experiences that matter to patients and families.

The PROM set used in this study reflects domains that are foundational aspects of an early palliative approach to care, namely communication quality, illness understanding, hope, emotional distress, and caregiving preparedness [16,55]. The high acceptability ratings suggest that patients and caregivers value structured opportunities to reflect on communication and their illness experience. This aligns with evidence that routine PROM collection can strengthen patient–clinician communication, enhance shared understanding, and empower patients in managing serious illness [21,56].

Although participants generally reported positive communication experiences and strong illness understanding, the PROMs revealed important nuances that are highly relevant to implementing a palliative approach to care. A meaningful minority believed cure was likely, underscoring persistent prognostic misperceptions even in settings assumed to be SIC competent. PROMs also highlighted substantial caregiver emotional burden and lower preparedness for the emotional aspects of caregiving. These findings are consistent with literature showing caregivers often experience greater distress than patients [53,54]. Qualitative comments emphasized needs related to coping with uncertainty, anticipatory grief, emotional support, and navigation challenges. These insights point to opportunities for guidance on anticipatory grief, emotional support, practical caregiving training, and navigation resources, which are interventions that align closely with early palliative care models.

This feasibility study also contributes to broader efforts to evaluate communication for serious illnesses. Prior evaluations of SIC and palliative care communication training have focused largely on clinician-reported outcomes (Kirkpatrick Levels 1–3), with limited assessment of real-world patient and caregiver outcomes [23]. By demonstrating that PROMs can be feasibly collected immediately following routine oncology encounters, our findings provide a potential methodological foundation for future trials evaluating communication training using Level-4 outcomes. PROMs capture communication quality and its consequences in ways that clinician-reported or simulated assessments cannot, offering a pathway to evaluate whether communication training meaningfully improves patient and caregiver experiences and supports earlier integration of palliative approaches.

The study has several limitations. Our findings show that one-time PROMs collection after an oncology visit was feasible in a selected and financially incentivized sample at one centre. While promising, more research is needed to determine the feasibility of large-scale implementation of these PROMs into routine practice, including consideration of clinician workload, electronic integration, perceived value of data, financial incentives, and other associated costs. PROM scores were skewed toward the high end, possibly reflecting the strong communication skills of participating oncologists, who may not represent typical practice. The PROMs should be further tested with a wider sample of clinicians who may have more varied experience in SIC, and repeated over time. Although the oncologists were encouraged to pre-screen and approach all eligible patients seen, we did not verify whether patients were excluded, which may have introduced selection bias. The survey being anonymous and the $50 gift card offered for completion, while certainly contributing to the high response rate achieved, may reduce the generalizability of our findings to routine clinical practice where financial incentives are not an option or identification of respondents is required. Other limitations include exclusion of caregivers not attending the appointment, possible digital literacy bias, lack of information about nonparticipants, ceiling effects, and the absence of longitudinal assessment. The single-centre design further limits generalizability, and anonymity prevented dyadic analyses between patient/caregiver pairs.

## 5. Conclusions

Patient and caregiver perspectives are essential for evaluating early palliative care and the communication practices that support this care. Our findings demonstrate that collecting PROMs online after routine oncology visits is feasible, acceptable, and informative, at least within the conditions of this study. Larger, multicentre longitudinal studies are needed to further assess feasibility in terms of PROM implementation into routine cancer care, repeated completion, responsiveness to change, and generalizability. Potentially, the PROMs we tested in this feasibility study can serve as objective intermediate and proximal indicators of communication quality, and as part of measures to assess the initiation of an early palliative approach to care. Applying this PROM set in pre- and post-intervention studies can help measure how effective SIC quality influences patient and caregiver outcomes over time, and in doing so, support earlier integration of palliative approaches in oncology care.

## Figures and Tables

**Table 1:** Patient and caregiver respondent characteristics.

	Patient (*N* = 72)		Caregiver (*N* = 65)	
	Number	Percent	Number	Percent
Gender—Female	38	52.8	38	58.5
Age range				
Under 30 years	0	0	1	1.5
30 to 39 years	2	2.8	4	6.2
40 to 49 years	6	8.3	4	6.2
50 to 59 years	11	15.3	15	23.1
60 years or older	53	73.6	41	63.1
Live with spouse or caregiver	54	75.0		
Cancer Diagnosis				
Lung	32	44.4		
Breast	10	13.9		
Colon and Rectal	6	8.3		
Esophagus	6	8.3		
Gynecological	6	8.3		
Liver	5	6.9		
Other	7	9.7		
Time since cancer diagnosis				
Less than 6 months	11	15.3		
6 months to less than one year	7	9.7		
1–5 years	43	59.7		
6 or more years	11	15.3		
Activity rating over past month of patient *				
Normal with no limitations	13	18.1	13	20.0
Not my normal self, but able to be up and about with fairly normal activities	35	48.6	34	52.3
Not feeling up to most things, but in bed or chair less than half the day	18	25.0	12	18.5
Able to do little activity & spend most of the day in bed or chair	5	6.9	4	6.2
Pretty much bedridden, rarely out of bed	1	1.4	2	3.1

* Rated by either patient or caregiver respondent.

**Table 2:** Study feasibility metrics.

Feasibility Metrics	Threshold for Success	Observed Value (Numerator/Denominator)
Recruitment (% of eligible patients that agree to consider completing the survey)	60%	93% (80 */86 *)
Rate of survey completion (% of all patients approached)	40%	83% (72/86 *)
Completeness of data collection in surveys	<10% missing data	<0.1% missing data
Spouse/caregiver representation (% of all spouses/caregivers approached who completed a survey)	50%	95% (65/68 *)
Willingness to repeat survey again in one month (% of respondents willing to complete the survey again)	50%	96% (69/72) (Caregiver 95% (62/65))
Willingness to repeat survey multiple times (% of respondents willing to complete the survey 3 times in the future)	30%	93% (56/72) (Caregiver 94% [61/65])
Willingness to have a conversation with oncologist recorded (% of respondents willing to have conversation with oncologist recorded)	30%	66% (47/71) (Caregiver 72% [47/65])

* Values are based on oncologist reporting and/or postcard distribution.

**Table 3:** Patient-reported outcome measures scores.

Patient-Reported Outcome Measures (PROMs)	Patient Reported Overall *N* = 72	Caregiver Reported Overall *N* = 65
Feeling Heard and Understood (FHU) scale [5-point scale, 0–4], Mean (SD)	3.9 (0.4)	3.9 (0.2)
Mean range, min–max (Median)	2.0–4.0 (4.0)	2.5–4.0 (4.0)
Item response indicated “4” = “Completely true”, % (number)		
I felt…		
(i) heard and understood by this provider and team	84.7 (61)	95.4% (62)
(ii) this provider and team put my best interests first when making recommendations about my care	91.7 (66)	98.5% (64)
(iii) the provider and team saw me as a person, not just someone with a medical problem	88.9 (64)	92.3% (60)
(iv) this provider and team understood what is important to me in my life	86.1 (62)	92.3% (60)
Herth Hope Index [4-point scale, 1–4], Mean (SD)	3.2 (0.4)	3.2 (0.4)
Mean Summary score [12,13,14,15,16,17,18,19,20,21,22,23,24,25,26,27,28,29,30,31,32,33,34,35,36,37,38,39,40,41,42,43,44,45,46,47,48] (SD)	38.6 (5.3)	37.8 (4.3)
Hospital Anxiety and Depression Scale (HADS) [4-point scale [0–3]] Total score on 7 items for each Anxiety and Depression	Anxiety 69.0% = Normal 14.1% = Borderline 16.9% = Abnormal	Anxiety 50.8% = Normal 32.3% = Borderline 16.9% = Abnormal
Normal = 0–7	Depression 77.5% = Normal 14.1% = Borderline 8.5% = Abnormal	Depression 64.6% = Normal 23.1% = Borderline 12.3% = Abnormal
Borderline = 8–10		
Abnormal = 11–21		
Preparedness for Caregiving Scale [caregivers only] [5-point scale, 0–4], Mean (SD)		2.6 (0.7)

SD = Standard deviation.

**Table 4:** Illness understanding item responses.

Illness Understanding Item *	Patient (*N* = 72)		Caregiver (*N* = 65)	
	Number	Percent	Number	Percent
1. For you, how true is the statement, I felt that my oncologist and their team respected my preferences for information about my diagnosis and treatment?				
Not true at all	0	0.0	0	0
A little bit true	0	0.0	0	0
Somewhat true	2	2.8	1	1.5
Very true	22	30.6	12	18.5
Completely true	48	66.7	52	80.0
2. All things considered, how do you feel about the amount of information you know about the likely outcome of your cancer over time (i.e., your prognosis)?				
I wish I had more information about my prognosis	10	13.9	10	15.4
I now have about the right amount of information	60	83.3	55	84.6
I wish I had less information about my prognosis	2	2.8	0	0
3. Patients differ in the amount of information that they want to know about their diagnosis and treatment—some want to know everything, others want to know very little. What is your preference for details of information about your diagnosis and treatment?				
I prefer not to hear a lot of details	2	2.8	1	1.5
I want to hear details only in certain situations, such as when tests are abnormal or when treatment decisions need to be made	17	23.6	9	13.8
I want to hear as many details as possible in all situations relating to my cancer and its treatment	53	73.6	55	84.6
4. How often have you had a conversation with your oncologist about the likely outcome of your cancer over time (i.e., your prognosis)?				
Never	1	1.4	8	12.3
Rarely	5	6.9	7	10.8
Sometimes	31	43.1	26	40.0
Often	28	38.9	19	29.2
Very often	7	9.7	5	7.7
5. Based on my understanding, my doctors believe the most likely outcome of my current treatment or care plan is:				
To cure the cancer completely	9	12.5	3	4.6
To slow down the spread of the cancer, though it cannot be cured	57	79.2	56	86.2
To focus on symptom management and comfort, as the cancer cannot be controlled any longer	6	8.3	6	9.2
6. Based on my understanding, my family believes the most likely outcome of my current treatment or care plan is				
To cure the cancer completely	11	15.3	7	10.8
To slow down the spread of the cancer, though it cannot be cured	55	76.4	55	84.6
To focus on symptom management and comfort, as the cancer cannot be controlled any longer	6	8.3	3	4.6
7. Based on my understanding, I believe the most likely outcome of my current treatment or care plan is:				
To cure the cancer completely	12	16.7	6	9.2
To slow down the spread of the cancer, though it cannot be cured	54	75.0	55	84.6
To focus on symptom management and comfort, as the cancer cannot be controlled any longer	6	8.3	4	6.2

* Items 2 to 4 taken directly from the Prognosis and Treatment Perceptions Questionnaire (PTPQ); items 5 to 7 adapted from the PTPQ.

## Data Availability

De-identified data are available from the corresponding author upon reasonable request.

## Abbreviations

The following abbreviations are used in this manuscript:

PROM	Patient-reported outcome measure
SIC	Serious illness communication
FHU	Feeling Heard and Understood scale
HADS	Hospital Anxiety and Depression Scale
